# Macrodomain ADP-ribose binding but not ADP-ribosylhydrolase activity is critical for chikungunya virus infection of *Aedes* mosquitoes

**DOI:** 10.1101/2025.10.09.681498

**Published:** 2025-10-09

**Authors:** Eugenia S. Bardossy, Lena Bergmann, Annabelle Henrion-Lacritick, Jared Nigg, Galen J. Correy, Alan Ashworth, James S. Fraser, Maria-Carla Saleh

**Affiliations:** 1 Viruses and RNAi Unit, Institut Pasteur, Université Paris Cité, F-75015 Paris, France.; 2 Department of Bioengineering and Therapeutic Sciences, University of California San Francisco, San Francisco, CA 94158.; 3 Helen Diller Family Comprehensive Cancer Center, University of California San Francisco, San Francisco, California, USA.

## Abstract

Viral macrodomains are promising antiviral targets that counteract host ADP-ribosylation-mediated antiviral responses in mammals. However, their role in dual-host viruses within the mosquito vector is largely unknown. Here, we investigated the role of the chikungunya virus (CHIKV) macrodomain by mutating the active site asparagine 24 (N24). In both mammalian and mosquito cell lines, these enzymes rapidly acquired compensatory mutations at aspartate 31 (D31). We show that while N24 mutations abolish ADP-ribosylhydrolase catalytic activity and reduce folding stability, ADP-ribose binding remains intact. Furthermore, the D31 compensatory mutations do not markedly rescue catalytic activity or folding stability. Structures of the compensatory mutant macrodomains suggest the importance of ADP-ribose binding, rather than ADP-ribosylhydrolase catalysis as the selective pressure driving their accumulation. In mammalian cells, viral mutants bearing the catalytic and compensatory mutations replicated less efficiently than wild-type virus in interferon-competent cell lines. However, their replication remained unaffected in mosquito cells. In *Aedes* mosquitoes, macrodomain mutations had disparate impacts, either reducing or enhancing infectivity and transmission depending on the specific mutation and viral lineage. These findings emphasize that viral macrodomain function is complex and host-dependent, highlighting the need for multi-host understanding to develop effective antivirals.

The global burden posed by emerging viruses, from pandemic coronaviruses to mosquito-borne arboviruses, requires innovative strategies to combat this threat. The identification of novel antiviral targets is essential to achieving this goal. Viral macrodomains — conserved protein domains found in RNA virus families — appear as potential candidates for antiviral drug discovery^1–8^, since they counteract the antiviral response of the infected cell. For instance, research on SARS-CoV-2, which encodes a viral macrodomain (Mac1), has led to the identification of promising compounds targeting this domain^9^. However, our understanding of the role of viral macrodomains during viral replication, particularly for dual-host viruses, is limited.

Viral macrodomains are encoded within the nonstructural genes of alphaviruses, coronaviruses, rubella virus, and hepatitis E virus^10,11^. They exhibit ADP-ribosylhydrolase catalytic activity, binding to mono- and/or poly-ADP-ribose (ADP-ribose) and removing it from ADP-ribosylated proteins^3–10^. Viral macrodomain activity is critical for viral RNA synthesis, replication and virulence^2–6,8,18–20^. Conversely, host cellular enzymes known as PARPs [polyadenosine diphosphate (ADP)-ribose polymerases] catalyze the addition of ADP-ribose moieties to target proteins. PARPs regulate key cellular processes and are major players in the mammalian antiviral defense^21,22^. Stimulated by interferon (IFN), some PARPs modulate IFN signaling^23–26^, while others directly suppress viral replication by modifying host or viral proteins^24,27^. In this cycle, viral macrodomains can “erase” what is “written” by PARPs^28^, turning the antiviral state into their favor, thereby promoting viral replication and pathogenesis^20,29,30^. Macrodomains can also have a “reader” function, binding to ADP-ribosylation marks on specific proteins. This activity is especially important for multidomain proteins, such as the human macrodomain containing PARP enzymes^31^, as it can serve to localize modified proteins to other catalytically active domains.

Some viral macrodomains are present in viruses that alternate between mammalian hosts and mosquito vectors, requiring adaptation to disparate cellular and physiological environments. Chikungunya virus (CHIKV), a re-emergent human pathogen transmitted by infected *Aedes* mosquitoes, exemplifies this challenge. The distinct immune responses and body temperatures of mosquito and mammalian hosts suggest that the established roles of viral proteins in one host may not directly translate to the other. For instance, mosquitoes rely on RNA interference rather than interferon for their antiviral response^32–35^. Therefore, the known function of viral macrodomains in counteracting the interferon-mediated antiviral response in mammalian cells may not apply during viral replication in mosquitoes. Furthermore, for the CHIKV macrodomain it remains unclear whether the “reader” or “eraser” activity is essential for viral fitness^36^.

CHIKV is an alphavirus in the *Togaviridae* family with a single-stranded, positive-sense RNA genome of approximately 11–12 kb. The genome contains two open reading frames (ORFs): the 5’ ORF, which is translated into four non-structural proteins (nsP1, nsP2, nsP3, and nsP4) that form the viral replicase complex; and the 3’ ORF, which encodes the structural proteins (capsid, E1, E2, E3, 6K, and TF) required for assembly of the mature virus particle^37^. The non-structural protein 3 (nsP3), an essential component of the viral replicase, is found in membrane-associated replication complexes and later in cytoplasmic granules. nsP3 has been also recognized as a determinant of mosquito vector specificity^38,39^ and mouse neurovirulence^4,40–44^. Notably, the catalytically active macrodomain is encoded within nsP3. While several studies have investigated the role of the alphavirus macrodomain during viral replication in mammalian cell culture and in mice, its role within the infected mosquito vector remains largely unknown.

Here, we investigated the role of the alphavirus nsP3 macrodomain during viral replication in both mammalian and mosquito hosts. Using CHIKV as a model, we engineered recombinant viruses carrying mutations in the macrodomain that eliminate its catalytic activity. Then, we investigated the effect of these mutations on viral replication in mammalian and mosquito cells. Compensatory mutations quickly evolved in both cell lines, but even with these additional mutations, the macrodomain remained catalytically inactive. However, none of these compensatory mutations affected binding affinity for ADP-ribose or stability. We assessed the role of the viral macrodomain in the mosquito vector *in vivo* for the first time by infecting laboratory colonies of *Aedes* mosquitoes with wild-type (WT) and mutant viruses. Our findings revealed that mutations in the macrodomain catalytic site could either reduce or enhance viral infectivity in the mosquito. Overall, we present a structural rationale for how a catalytically dead viral macrodomain can play a role in the fitness of an alphavirus in mosquitoes.

## Results

### Asparagine 24 is not conserved in the macrodomain of mosquito-specific alphaviruses

**Figure 1.**
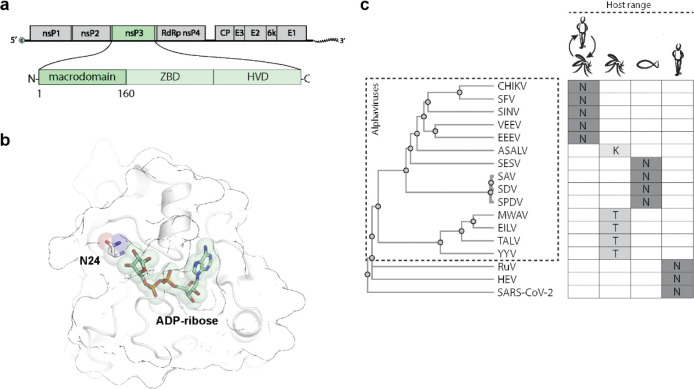
Asparagine 24 (N24) is not conserved in the macrodomains of mosquito-specific alphaviruses. **a**, The CHIKV macrodomain is encoded within the first 160 amino acids of the viral nsP3 gene. **b**, Structure of the CHIKV macrodomain highlighting asparagine 24 (N24) and bound ADP-ribose (PDB 3GPO). **c**, Conservation analysis of residue 24 in macrodomains from 14 alphaviruses with different host tropisms: dual-host (CHIKV, SFV, SINV, VEEV, EEEV), mosquito-specific (ASALV, MWAV, EILV, TALV, YYV), and aquatic (SESV, SAV, SDV, SPDV). Macrodomain sequences from other human-pathogenic viruses (RuV, HEV, SARS-CoV-2) were also included. Protein sequences were obtained from the PDB or GenBank. Multiple sequence alignment (see Supplementary Figure 1a) and phylogram construction were performed using Clustal Omega tools. ZBD: zinc-binding domain; HVD: hypervariable domain; N: asparagine; K: lysine; T: threonine.

The nsP3 of alphaviruses comprises three major domains (Fig. 1a): a highly conserved N-terminal macrodomain, a central zinc-binding domain, and a C-terminal hypervariable region. In CHIKV, the macrodomain spans the first 160 amino acids of nsP3. Sequence and structure comparisons across cellular and viral macrodomains have highlighted highly conserved residues and motifs^1,5,7,12,17,18,30,45–52^. Further mutagenesis studies have identified specific conserved residues that are critical for catalytic activity, including those that are essential for ADP-ribose binding and/or hydrolase activity^2–5,13,16,17,47^. Among them, asparagine 24 (N24) of the CHIKV macrodomain (Fig. 1b) has been established as pivotal for maintaining catalytic function^2,3,13,16,17^ and is reportedly 100% conserved among macrodomains of human-pathogenic viruses.

To investigate N24 conservation across alphaviruses, we examined its prevalence in viruses infecting diverse hosts. Based on host tropism, alphaviruses can be classified as: (i) dual-host viruses that alternate between vertebrates and mosquitoes; (ii) aquatic alphaviruses, infecting non-mosquito hosts; and (iii) mosquito-specific alphaviruses, incapable of infecting vertebrates. We compared macrodomain protein sequences from 14 alphaviruses representing these groups, also including SARS-CoV-2, rubella virus, and hepatitis E virus macrodomains (Supplementary Fig. 1a and 1b). Our analysis revealed 100% N24 conservation in macrodomains of dual-host and aquatic alphaviruses, as well as in the other human-pathogenic viruses (Fig. 1c). However, N24 was not conserved in macrodomains of mosquito-specific alphaviruses. This observation suggests that the macrodomain of dual-host alphaviruses is subject to different selective pressures in their alternating hosts. While a fully active macrodomain may be essential for replication in vertebrates, it appears to be unnecessary for replication in only mosquitoes. Based on this finding, we hypothesize that mutations in alphavirus’s macrodomain catalytic site may not affect viral replication in mosquitoes.

### CHIKV N24 macrodomain mutants select for compensatory mutations in residue 31 during viral replication in mammalian and mosquito cells

**Figure 2.**
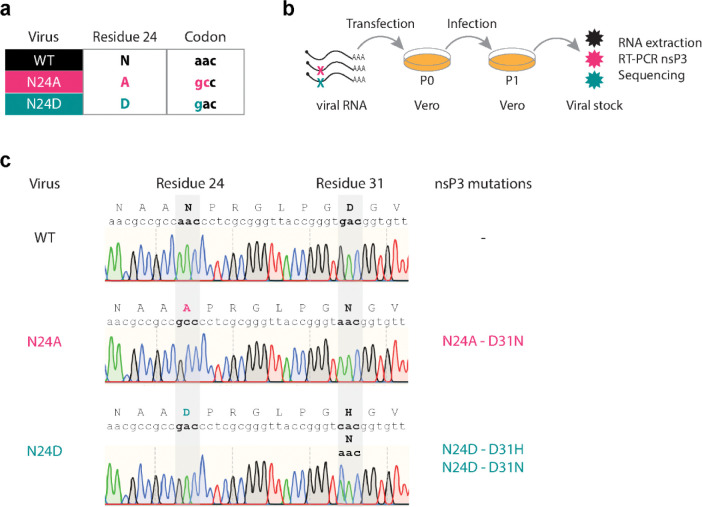
N24 mutations in the CHIKV macrodomain lead to the emergence of secondary mutations in D31 during viral stock production. **a**, Point mutations were introduced into the CHIKV infectious clone in the viral macrodomain to change residue 24 from wild-type asparagine (N) to alanine (A) or aspartic acid (D) to generate two mutant viruses, N24A and N24D, respectively. **b**, For viral stock production, *in vitro* transcribed viral RNAs for WT, N24A, or N24D were transfected into Vero cells. Cell supernatant was collected after transfection (P0) and used to infect fresh Vero cells. The supernatant from this passage (P1) was then subjected to RNA extraction, RT-PCR amplification of the nsP3 gene, and Sanger sequencing. **c**, Sanger sequencing chromatograms of WT, N24A, and N24D viruses from P1. N24A and N24D sequences show novel mutations in residue 31 (D31N and D31H).

To test our hypothesis, we designed CHIKV recombinant viruses harboring mutations in N24 macrodomain catalytic site and studied viral replication of WT and mutant viruses in mammalian and mosquito cell lines. Specifically, we introduced N24A and N24D point mutations into the nsP3 gene of a CHIKV infectious clone (Fig. 2a). The N24A and N24D mutations (asparagine to alanine or aspartic acid substitution, respectively) have been previously demonstrated to abolish macrodomain activity in coronaviruses^2,12^. The homologous mutation to N24A in SARS-CoV-2 Mac1 is thermodynamically destabilized^2^ and we have therefore instead preferred to use the N24D (alanine to aspartic acid substitution) mutation, which is also catalytically inactive, but is as stable as WT in SARS-CoV-2^2^. Nonetheless, we introduced both mutations into the CHIKV macrodomain. First, we assessed the stability of the introduced mutations during viral stock production in mammalian cells (Fig. 2b). For this, equal amounts of RNA transcripts for WT, N24A, or N24D viruses were transfected into Vero cells. After three days, cell culture supernatants (P0) were collected and used to infect fresh cells (P1). Total RNA was extracted from P1 culture supernatants and used as a template for reverse transcriptase reactions. From cDNA, the nsP3 viral gene was amplified by PCR, and the purified products were sent for Sanger sequencing. We confirmed the presence of the introduced N24A and N24D mutations in the P1 viral stocks of the mutant viruses (Fig. 2c). However, secondary mutations in residue 31 of the nsP3 gene had arisen in both mutant viruses. Residue 31 changed from D to N (aspartic acid to asparagine substitution) in the N24A mutant virus, and to H or N (aspartic acid to histidine or asparagine substitution) in the N24D mutant virus (Fig. 2c). To assess the dynamics of the emergence of D31 mutations, we sequenced virus stocks recovered from earlier time points (Supplementary Fig. 2a). Our time-course sequence analyses revealed the presence of D31 mutations as early as one day after RNA viral transfection in both mutant viruses. Importantly, no secondary mutations emerged in the WT stock (Supplementary Fig. 2a). These findings suggest that the N24A and N24D mutations are deleterious for CHIKV replication in Vero cells. Secondary mutations in residue 31 arise quickly during the initial replication cycles to rescue the viral population.

To investigate if the emergence of the D31 mutation depends on the host, we produced viral stocks in various mammalian (A549, BHK-21, and Vero) and mosquito (C6/36) cell lines. Following transfection with WT, N24A, and N24D viral RNAs, cell culture supernatants were harvested at various time points after transfection (P0) and after one viral passage (P1) (Supplementary Fig. 2b). Then, total RNA was extracted and processed as previously described. For the N24A mutant virus, the D31N mutation emerged in BHK-21, Vero, and C6/36 cells at different time points. In Vero cells, D31N mutation arose two days after transfection, and rapidly displaced the WT residue in position 31. Conversely, in BHK-21 and C6/36 cells, the D31N mutation emerged in passage one, where a mixture of single N24A and double N24A-D31N mutant viruses was detected. For the N24D mutant virus, the D31N mutation emerged in Vero and BHK-21 cells, coexisting with the parental sequence as a mixture of viruses. Notably, in this experiment, partial reversions of the introduced N24 mutations occurred for both mutants. N24A partially reverted to the WT sequence in A549 cells after one passage, and N24D partially reverted to the WT sequence in the three mammalian cell lines at different time points (Supplementary Fig. 2b). Again, no secondary mutations emerged in the WT virus in any of the tested cell lines. Overall, our sequence analyses demonstrate that secondary mutations emerge at residue 31 of the viral macrodomain when parental viral genomes with primary mutations in the catalytic N24 site replicate in both mammalian and mosquito cell lines.

Next, we investigated the emergence of D31 mutations in the context of a CHIKV strain from a different lineage. There are three main CHIKV lineages: African, East-Central-South African (ECSA), and Asian. Our previous experiments were done with the Caribbean strain from the Asian lineage, which was responsible of the introduction of CHIKV in the Americas. We went on to introduce N24A and N24D mutations into an infectious clone of the Indian Ocean strain, a representative of the ECSA lineage responsible for the 2005 outbreak in La Reunion. As before, we assessed the genetic stability of N24 mutations and the potential emergence of secondary mutations in the macrodomain during viral stock production. We observed that the D31N secondary mutation consistently emerged in both N24A and N24D Indian Ocean mutant viruses during viral stock generation in mammalian cells (Supplementary Fig. 2c). Taken together, our results show that CHIKVs with single N24A or N24D mutations are not viable in mammalian or mosquito cell lines, independent of the viral lineage. In this scenario, viral replication is restored by the emergence of compensatory mutations at residue 31 and, in some cases, by partial reversion of the N24 mutation.

### N24 mutations abolish macrodomain catalytic activity *in vitro* while retaining ADP-ribose binding

**Figure 3.**
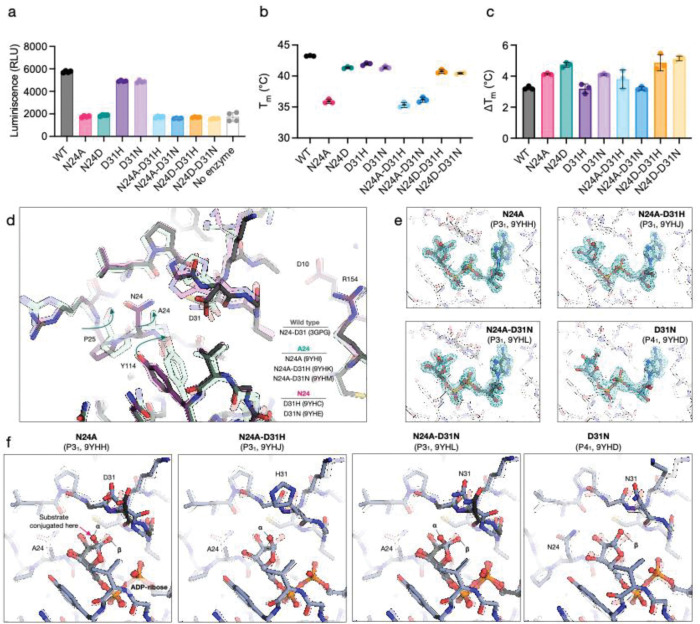
Functional and structural characterization of CHIKV nsP3 macrodomain mutants. **a**, Assays of the ADP-ribosylhydrolase activity of recombinantly expressed wild-type (WT) and mutant macrodomains. Macrodmains (200 nM) were incubated with auto-MARylated human PARP10 for 1 hour at room temperature and the production of ADP-ribose was measured using NUDT5 and an AMP-Glo luciferase assay^2^. Data are plotted mean ± SD for four technical replicates. **b**, Thermostability of CHIKV nsP3 macrodomain mutants measured by DSF using SYPRO orange. Data are plotted mean ± SD for three technical replicates. **c**, Change in CHIKV nsP3 macrodomain thermostability upon incubation with 1 mM ADP-ribose. **d,** Alignment of the crystallographic structures of WT, N24A, N24A-D31N, N24A-D31H, D31H and D31N reveals a peptide flip in P25 and a coupled shift in Y114 in structures containing the N24A mutation. For clarity, only chain A is shown (see Supplementary Fig. 4a for all chains). **e**, Difference electron density maps (F_O_-F_C_, contoured at 3 σ) calculated prior to modeling ADP-ribose. Maps for all chains are shown in Supplementary Fig. 5. **f**, Crystal structures of N24A, D31N, N24A-D31H and N24A-D31N bound to ADP-ribose. Chain A is shown for the P3_1_ structures and chain D for the P4_1_ structure. Although the ADP-ribose binding pose is conserved, there is a rotameric shift at residue 31 that accompanies ADP-ribose binding, and mutations at position 31 change the character of the exit path of the substrate suggesting a role for substrate-specific recognition.

Our attempts to produce viral stocks with single N24 mutations in the CHIKV macrodomain resulted in the repeated recovery of double mutants containing the parental N24 mutation plus the new D31 mutations. Therefore, we decided to investigate the structural and functional contributions of N24 and D31 to macrodomain binding and catalytic activity *in vitro*. To this end, we constructed expression plasmids encoding WT and mutant versions of the CHIKV macrodomain. We purified the following recombinant macrodomains variants from bacteria: WT, N24A, N24D, D31H, D31N, N24A-D31H, N24A-D31N, N24D-D31H, and N24D-D31N (see Supplementary Fig. 3a). First, we assessed the catalytic activity of recombinant macrodomains using an AMP-Glo luciferase assay^2^ (Fig. 3a). All N24 mutants (in either single or double combinations with D31) were catalytically inactive, exhibiting equivalent luciferase activity to the non-enzyme control. In contrast, single D31 mutants did not significantly alter catalytic activity compared to the WT, suggesting that the selective pressure that led to their accumulation was not related to catalytic activity.

Next, we determined the thermostability and ADP-ribose binding function of the mutants using differential scanning fluorimetry (DSF) (Fig. 3b,c, Supplementary Table 1, Supplementary Fig. 3b). This analysis revealed that the N24A mutant is less stable (ΔT_m_ relative to WT = −7.3°C) than the N24D mutant (ΔT_m_ relative to WT = −1.9°C), paralleling results with the SARS-CoV-2 macrodomain^2^. Both mutants also retained ADP-ribose binding, generating a similar T_m_ shift in the presence of 1mM ADP-ribose to WT (3.3°C for WT, 4.1°C for N24A, 4.7°C for N24D) (Fig. 3c, Supplementary Table 1). The compensatory mutations had minimal effects on both stability and ADP-ribose binding function. The D31 mutations were neutral in the WT background and did not rescue stability in the N24-mutant backgrounds. This result suggests that although N24A is destabilizing, the compensatory mutations do not act to restore thermodynamic stability.

To examine how these mutations might alter structure and function, we determined X-ray structures of N24A, N24A-D31H, N24A-D31N, D31H and D31N (Fig. 3d, Supplementary Table 2). The structures reveal a shift in residues 24–25 in the N24A mutants, with a flip in the P25 carbonyl and a correlated shift in nearby Y114 side chain (Fig. 3d, Supplementary Fig. 4a,b). Given the lack of change in either catalytic or ADP-ribose binding relative to the N24 mutations alone, it is expected that the compensatory residues do not alter the interactions with ADP-ribose. To test this, we soaked ADP-ribose into crystals and determined structures of N24A, N24A-D31H, N24A-D31N and D31N (Fig. 3e, Supplementary Table 2, Supplementary Fig. 5). The structures show that the D31 mutants undergo a similar side chain rotation when ADP-ribose binds (Fig. 3f). We observed both the α and β anomers of ADP-ribose bound (Fig. 3f, Supplementary Fig. 4c–f): the α anomer is consistent with the substrate bound state, while both anomers are consistent with product bound^53^. The D31 mutations line the “exit” path of the residue modified with ADP-ribose, leaving them poised to influence substrate binding to the macrodomain (Fig. 3f). Collectively, our results suggest that the “eraser” catalytic activity of the macrodomain is dispensable for function, but that “reader” ADP-ribose recognition is likely required. The conserved location of independently acquired compensatory mutations suggests that they may have been selected to tune the “reader” activity towards specific substrates. With this information, we went on to evaluate the role of the CHIKV macrodomain during viral replication in different hosts.

### Mutations in N24 affect viral replication and potential of transmission in CHIKV infected *Aedes* mosquitoes

**Figure 4.**
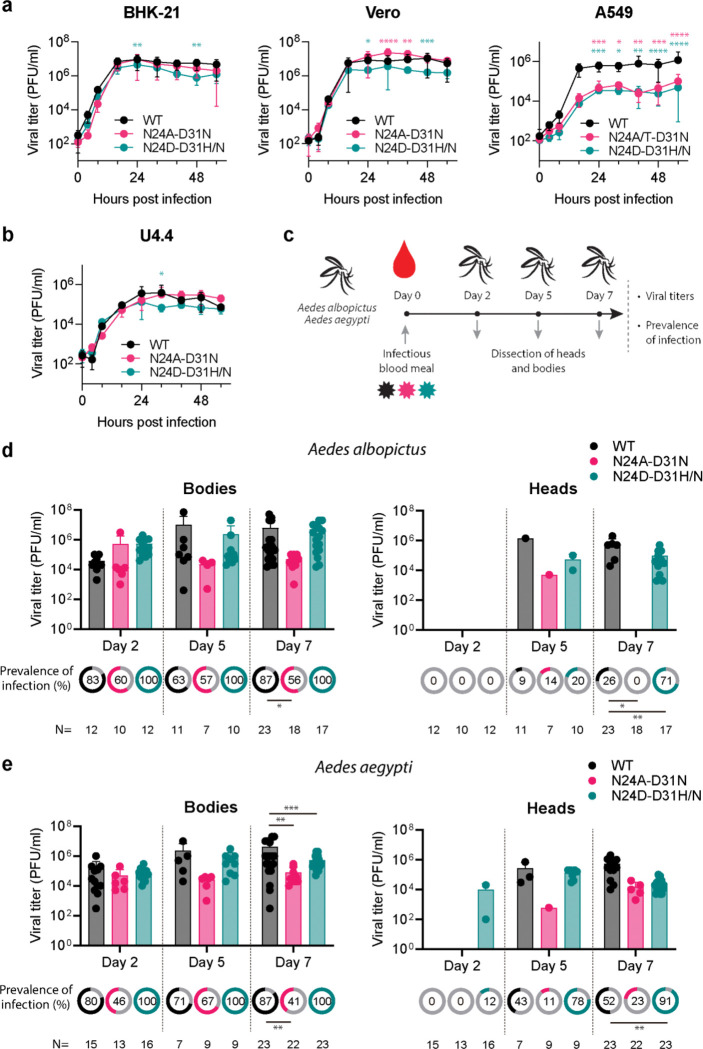
Effect of mutations in the macrodomain on CHIKV replication in mammalian and mosquito cells and in *Aedes* mosquitos. **a**, Growth kinetics of Caribbean WT and mutant viruses (N24A-D31N and N24D-D31H/N) in BHK-21, Vero, and A549 mammalian cells, and in **b**, mosquito U4.4 cells. Error bars represent standard deviations from the mean. n = 3 for 0, 4, 8, 16, 32, 40 and 56 hours post infection (hpi), and n = 6 for 24 and 48 hpi. Data were analyzed using a mixed-effects model with the Geisser-Greenhouse correction followed by a Tukey’s multiple comparison test. Pink and blue asterisks indicate p-values in comparison to WT. **c**, Schematic representation of the in vivo experiments for infecting mosquitoes. Laboratory colonies of *Aedes albopictus* and *Aedes aegypti* were exposed to a blood meal containing WT, N24A-D31N (in pink) or N24A-D31H/N (in blue) viruses. After 2, 5 and 7 days, individual mosquitoes were collected for dissection. Plaque assays were performed on heads and bodies. **d**, Viral titers and prevalence of infection in the bodies (left panel) and heads (right panel) of *Ae. albopictus* and **e**, *Ae. aegypti* mosquitoes. The number of mosquitoes analyzed is indicated below (N). Error bars represent standard deviations from the mean. Viral titer data were analyzed using a two-way ANOVA followed by a Tukey’s multiple comparison test. Prevalences were compared using Fisher’s exact test.

We studied the effect of macrodomain mutations in CHIKV viral replication in its different hosts. First, we assessed the replication of the Caribbean WT and double mutant (N24A-D31N and N24D-D31H/N) viruses by growth curves in mammalian and mosquito cell lines (Fig. 4a and 4b). In mammalian cells lacking a competent interferon response, such as BHK-21 and Vero cells, the N24A-D31N and N24D-D31H/N mutant viruses replicated similarly to the WT virus (Fig. 4a). However, in human A549 cells, which mount a normal interferon response, both mutant viruses reached significantly lower viral titers than the WT virus at several time points after infection (Fig. 4a). This finding is consistent with the established notion that viral macrodomains counteract the infected cell’s antiviral response: when the macrodomain activity is hindered, viral replication is impaired. In *Ae. albopictus* U4.4 cells, the replication kinetics of the Caribbean WT and mutant viruses were similar (Fig. 4b). This result suggests that a catalytically active macrodomain is unnecessary for CHIKV replication in mosquito cells. Next, we examined the impact of the N24-D31 mutations in the context of the Indian Ocean CHIKV strain (Supplementary Fig. 6a). In BHK-21 cells, mutant viruses N24A-D31N and N24D-D31N replicated similarly to the WT. In Vero cells, N24D-D31N reached lower viral titers than WT at 24, 40, and 56 hours post-infection. In human A549 cells, both mutant viruses reached lower viral titers than the WT, with a stronger attenuation effect observed for N24D-D31N. Conversely, in U4.4 mosquito cells, the N24D-D31N virus reached viral titers slightly slower than the WT virus, though the difference was not significant (see Supplementary Fig. 6b). Of note, the presence of both the N24 and D31 mutations in growth curve samples (collected at 48 hours for the Caribbean strain and 72 hours for the Indian Ocean strain) was confirmed by nsP3 RT-PCR and Sanger sequencing (Supplementary Fig. 7a and 7b). Overall, our experiments in cell culture show that CHIKV macrodomain catalytic activity is important for replication in interferon-competent mammalian cells but not in mosquito cells.

Next, we sought to investigate the *in vivo* role of the viral macrodomain in the mosquito host. While studies in mice have examined the function of alphavirus macrodomains in mammalian hosts^3,41,44^, no such studies have been conducted in mosquitoes. To this end, we exposed laboratory colonies of *Ae. albopictus* and *Ae. aegypti* mosquitoes to an infectious blood meal containing Caribbean WT, N24A-D31N, or N24D-D31H/N viruses (Fig. 4c). At two, five-, and seven-days post-infection, blood-fed individual mosquitoes were collected and dissected. Plaque assays were performed on the heads and bodies to determine their infectious status (infected or uninfected). The presence of the virus in the body indicates its ability to efficiently infect and disseminate within the mosquito, while viral presence in the head reflects the potential for viral transmission through saliva. This experimental design allowed us to assess two key parameters: prevalence of infection (percentage of infected heads and bodies) and viral titer (level of viral replication in infected mosquitoes).

In *Ae. albopictus* mosquitoes, viral titers in bodies were comparable between WT and mutant viruses at all time points examined (Fig. 4d). However, at day 7, the percentage of infected mosquito bodies was significantly lower for the N24A-D31N mutant compared to the WT virus. Conversely, the N24D-D31N mutant exhibited 100% infection prevalence in mosquito bodies at all time points, whereas WT prevalence ranged from 63% to 87%. Viral presence in the heads of *Ae. albopictus* mosquitoes was detected only on days 5 and 7. While a comparison of viral titers in heads was not possible due to the limited number of infected heads, a notable difference in head infection prevalence was observed on day 7: 26% for WT, 71% for the N24D-D31N mutant, and 0% for the N24A-D31N mutant. In *Ae. aegypti*, viral titers in bodies were significantly lower for both mutant viruses than for WT on day 7 (Fig. 4e). Consistent with the findings in *Ae. albopictus*, the prevalence of infection in the bodies of N24A-D31N-infected *Ae. aegypti* mosquitoes was significantly lower than in the WT on day 7. Again, N24D-D31N-infected bodies showed 100% prevalence. In *Ae. aegypti* heads, the prevalence of N24D-D31N at day 7 was 91%, significantly higher than the 51% prevalence observed for WT. Taken together, these results suggest that inactivating the CHIKV macrodomain can have distinct effects on viral replication in mosquitoes, depending on specific mutations present in the nsP3 gene. While the N24A-D31N mutation impairs viral dissemination in mosquitoes, the N24D-D31H/N mutation enhances it, compared to the WT virus.

To evaluate if the *in vivo* effects of macrodomain mutations depend on the CHIKV strain, we infected mosquitoes with the Indian Ocean WT and mutant viruses (Supplementary Fig. 6c and 6d). In *Ae. albopictus*, we observed significant differences at day 5 post-infection: mutant viruses reached significantly higher viral titers in mosquito bodies and higher head infection prevalences compared to the WT virus. In infected *Ae. aegypti*, we found no differences in viral titers and prevalences of infections between WT and mutant viruses. Notably, experimental infection of *Ae. aegypti* with IOL viruses was highly efficient, with 100% of mosquito bodies infected across all tested time points, which likely masked potential variations between WT and mutant viruses. Of note, RT-PCR and sequencing of nsP3 confirmed the presence of both the introduced N24 and the secondary D31 mutations in viral RNA from individual infected *Ae. aegypti* and *Ae. albopictus* mosquitoes from both the Caribbean and IOL infection groups (see Supplementary Table 3).

Overall, our cell culture and *in vivo* experiments reveal the complex role of an active CHIKV macrodomain in viral replication and transmission. While macrodomain activity is crucial for viral replication in interferon-competent mammalian cells, it appears less critical in mosquito cells. In *Aedes* mosquitoes, the impact of macrodomain mutations varies depending on the specific mutation and viral strain, with some mutations impairing viral dissemination (N24A-D31N) and others enhancing transmission potential (N24D-D31H/N). These findings highlight the strain-specific and context-dependent nature of viral macrodomain functions in dual-host viruses.

## Discussion

Here, we investigated the requirement of a catalytically active macrodomain for viral replication and transmission in dual-host viruses, using CHIKV as a model. By introducing specific mutations (N24A and N24D) into the conserved catalytic asparagine N24 of the CHIKV nsP3 macrodomain, we successfully abolished its ADP-ribose hydrolase activity while preserving substrate binding. However, recombinant CHIKV strains carrying N24 mutations rapidly acquired compensatory D31 secondary mutations in several cell lines. Even with these compensatory mutations, N24-mutant strains exhibited impaired replication in interferon-competent mammalian cells. However, all strains replicated comparably to WT in interferon-deficient mammalian and mosquito cells. More importantly, our *in vivo* studies in *Ae. aegypti* and *Ae. albopictus* mosquitoes provide the first detailed evidence that macrodomain mutations significantly modulate viral infectivity and transmission potential in the natural vector, with effects varying depending on the specific mutation and viral lineage.

The fact that CHIKV, like other alphaviruses, jumps between vertebrate and invertebrate hosts introduces unique evolutionary pressures on its viral proteins. Our findings, consistent with other reports, reveal a striking loss of macrodomain activity in insect-specific alphaviruses^48,54^. This observation strongly suggests that a catalytically active macrodomain is less critical, or even dispensable, for replication in the mosquito vector, a hypothesis that guided our initial investigations. However, our experimental data also present a more nuanced picture, showing that while some N24 mutations impair viral dissemination in mosquitoes (N24A-D31N), others can enhance it (N24D-D31N). This complexity is further highlighted by the existence of at least two alphaviruses with reported loss-of-function macrodomains that still infect vertebrates^48^.

Are we fully appreciating the repertoire of macrodomain functions beyond their enzymatic activity? Moreover, is our understanding of the viral macrodomain role biased because most previous studies were conducted in mammalian systems, where ADP-ribosylation is key in mounting the antiviral response? The varied outcomes of macrodomain inactivation in our study, from impaired replication in interferon-competent mammalian cells to differential effects in mosquitoes, suggest that the macrodomain may possess yet-to-be-understood roles that contribute to viral fitness in a context-dependent manner. Our work suggests that we may currently underappreciate the “reader” function of macrodomains and that the compensatory mutations may work to bias certain modified proteins to interact with nsP3. The importance of this kind of scaffolding function of macrodomains may be related to the higher order structures formed by nsP3 that are needed to interact with host proteins^55^ or organize tubular structures that contain viral genomic RNA^56^. Ultimately, a deeper understanding of these intricate macrodomain functions is needed to develop new therapeutic strategies that can effectively disrupt the viral replication cycle.

## Methods

### Sequence alignment and phylogram construction

Protein sequences of the nsP3 macrodomain from 14 alphaviruses and 3 other human viruses were downloaded from GenBank and the Protein Data Bank (PDB). The protein sequence identifiers and full names of the viruses are detailed in Supplementary Figure 1B. Multiple sequence alignment and phylogenetic tree construction were performed using the online Clustal Omega tools^57^.

### Construction of recombinant viruses

To introduce the N24A and N24D mutations into the nsP3 gene of the CHIKV-Caribbean^58^ and - Indian Ocean^59^ infectious clones, 5’-phosphorylated primers were used to amplify the full-length plasmid template via inverse PCR using Q5 High Fidelity DNA Polymerase (New England Biolabs). Forward primers contained 1–2 mismatches relative to the WT CHIKV sequences to incorporate the desired nucleotide changes into the PCR product. N24A mutations were introduced using the forward primer 5’-GACCCTCGCGGGTTACCG-3’ for both infectious clones. N24D mutations were introduced using the forward primer 5’-GCCCCTCGCGGGTTACCG-3’ for both infectious clones. Reverse primer for CHIKV-Caribbean was 5’-GGCGGCGTTGACCACGCA-3’ for both mutants, and reverse primer for CHIKV-Indian Ocean was 5’-AGCGGCGTTGACTACGCA-3’ for both mutants. Purified PCR products were then blunt-end ligated with T4 DNA ligase and transformed into competent *E. coli* cells. Colonies were miniprepped, and plasmid sequences were verified by whole-plasmid sequencing (Eurofins).

The DNAs of the recombinant constructs were linearized by digestion with NotI enzyme and used as templates for transcription by SP6 polymerase in the presence of GpppG cap structure analog, using the mMessage mMachine transcription kit (Thermo Fisher). The RNAs were gel-quantified and used for transfection in cell culture.

### Cells, viral transfections and infections

Mammalian cell lines (Vero, BHK-21, and A549) were maintained in Dulbecco’s Modified Eagle Medium (D-MEM) supplemented with 10% fetal bovine serum (FBS) and 1% penicillin/streptomycin (P/S) at 37°C with 5% CO_2_. Ae. albopictus cell lines (C6/36 and U4.4) were maintained in Leibovitz L-15 medium supplemented with 10% FBS, 1% P/S, 1% non-essential amino acids, and 1% tryptose phosphate broth at 28°C with 5% CO_2_.

For virus stock production, Vero (for Caribbean strain) or BHK-21 (for Indian Ocean strain) cells were grown to 60–70% confluence in T75 flasks and transfected with 10 ug of in vitro transcribed viral RNAs using Lipofectamine 2000 (Invitrogen). Two to three days post-transfection, cell culture supernatants were used to infect fresh Vero cells. Viral stocks were harvested from cell culture supernatants 2–3 days after infection and quantified by plaque assay.

### Plaque assays

Viruses from culture supernatants were quantified by plaque assays to determine viral yields. Vero cells (1 × 10^5^ per well) were seeded into 24-well plates and allowed to attach overnight. Viral stocks were serially diluted, and 0.1 mL was added to the cells, followed by a 1-hour incubation. An overlay medium (1× DMEM, 2% FBS, 1% P/S, and 0.8% agarose) was then added to each well. Cells were fixed three days post-infection with 4% paraformaldehyde and stained with crystal violet for plaque visualization.

### Assessment of mutations in viral macrodomains

Mammalian (Vero, BHK-21, and A549) and mosquito (C6/36) cells were transfected in T25 flasks with 3 μg of in vitro transcribed viral RNAs as previously described. Cell culture supernatants were harvested at various time points post-transfection for total RNA extraction, nsP3 RT-PCR amplification, and Sanger sequencing. Viruses recovered from supernatants were used to re-infect fresh cells, and cell culture supernatants from viral passages were also harvested for total RNA extraction, nsP3 RT-PCR, and sequencing.

Viral RNAs were extracted from culture supernatants using TRIzol (Invitrogen) and used for reverse transcription (RT) reactions with oligo-dT primers (Maxima H Minus RT, Thermo Fisher Scientific). PCR reactions were performed using primers forward 5’-GCAGTTTTGACAATGGCAGAAG-3’ and reverse 5’-TGTCTTTCCCTCCTGAGTATACAC-3’ (DreamTaq DNA Polymerase, Thermo Fisher Scientific). The size and quality of the amplicons were assessed by electrophoresis on a 1.5% agarose gel. The 453-bp amplicons were cleaned up using ExoSAP-IT^™^ PCR Product Cleanup Reagent (Thermo Fisher Scientific) and submitted for Sanger sequencing (Eurofins).

For each PCR sample, individual Sanger chromatograms were visually inspected to assess sequence quality and detect double peaks. All high-quality sequences were aligned against the wild-type (WT) CHIKV nsP3 gene to identify reversions of introduced mutations and detect new mutations.

### Protein purification

Plasmid DNA (pET22b(+)) encoding His_6_-tagged wild type or mutant (N24A, N24D, D31H, D31N, N24A-D31H, N24A-D31N, N24D-D31H, N24D-D31N) macrodomains were transformed into BL21 (DE3) *Escherichia coli*. After overnight growth at 37°C (LB-agar, 100 μg/ml carbenicillin), single colonies were used to inoculate small scale cultures (10 ml LB, 100 μg/ml carbenicillin). Following overnight growth at 37°C, large scale cultures (1 l LB, 100 μg/ml carbenicillin) were inoculated and grown at 37°C until an OD600 = 0.8, at which point protein expression was induced with 0.2 mM IPTG at 18°C over night. Cells were harvested by centrifugation (4000 g, 20 min, 4°C) and cell pellets were briefly washed with phosphate-buffered saline before being re-centrifuged (4000 g, 20 min, 4°C). The cell pellets were frozen at −80°C until further purification. Thawed cell pellets were resuspended in 25 ml Lysis buffer (50 mM Tris pH 8.0, 300mM NaCl, 20mM imidazole, 5% glycerol, 0.1% Triton X100) and 1/2 tablet of protease inhibitor (EDTA-free cOmplete, Roche). Cells were lysed by sonication (2 min) and cell debris was collected by centrifugation (30,000 g, 30 min, 4°C). The supernatant was filtered (0.45 μm cellulose acetate) and loaded on a 5 ml HisTrap HP column pre-quilibrated with buffer A (50 mM Tris pH 8.0, 150mM NaCl, 20mM imidazole). Bound protein was eluted via a linear gradient of buffer B (50 mM Tris pH 8.0, 150mM NaCl, 300mM imidazole). Eluted protein was pooled, concentrated and purified via size exclusion chromatography (HiLoad 16/600 Superdex 75, Cytiva) in 10 mM Tris pH 8.0 and 300 mM NaCl. Relevant fractions were pooled, concentrated and stored at −80°C.

### AMP-Glo assay

The ADP-ribosyl hydrolase activity of CHIKV nsP3 macrodomain wild type and mutants was determined using auto-MARylated human PARP10 as a substrate following the method described previously (PMID: 37651466). ADP-ribose produced by the macrodomain is hydrolyzed by NUDT5 phosphodiesterase to AMP, which was detected using the commercially available AMP-Glo kit (Promega, V5011). Reactions were prepared to a final volume of 8 μl in assay buffer (20 mM HEPES pH 7.5, 100 mM NaCl, 10 mM MgCl_2_) and contained 100 nM NUDT5, 10 μM MARylated PARP10 and 200 nM CHIKV nsP3 macrodomain or buffer. The AMP concentration was quantified using a BioTek Synergy H1 plate reader after incubation of the reactions at room temperature for 1 hour. Enzyme concentration was calibrated so that no more than 20% of the substrate was consumed during the reaction. All reactions were performed in technical quadruplicates. Data was plotted using GraphPad Prism.

### Crystallography

CHIKV nsP3 macrodomain mutants were crystallized using hanging drop vapor diffusion and MRC 2-well crystallization plates. Crystals grew at room temperature from drops contained 200 nl protein and 200 nl reservoir. Crystallization conditions are reported in Supplementary Table 2. To obtain structures of mutants bound to ADP-ribose, crystals were soaked in reservoir solutions supplemented with 20 mM ADP-ribose. Crystals were vitrified in liquid nitrogen without additional cryoprotection. Data were collected at 100 K using beam line 8.3.1 of the Advanced Light Source. Data were indexed, integrated and scaled with XDS^60^ and merged with Aimless^61^. Phases were obtained by molecular replacement run with Phaser^62^ using chain A from PDB 3GPG as a search model. Initial models were improved by iterative cycles of model building using COOT^63^ and refinement with phenxi.refine^64^. During initial rounds of refinement, waters were automatically added using phenix.refine. During final rounds of refinement, hydrogens were added to protein coordinates using phenix.ready_set and refined using a riding model. B-factors were refined anisotropically for non-hydrogen atoms except for the structure of the D31N mutant that crystallized in P4_1_ (PDB 9YHD). Coordinates and restraints for ADP-ribose were generated with phenix.elbow^65^. Data collection and refinement statistics are summarized in Supplementary Table 2.

### DSF

Differential scanning fluorimetry (DSF) was performed to determine the thermal stability of the target protein using SYPRO Orange dye (Thermo Fisher Scientific. The protein was diluted to a final concentration of 3 μM in assay buffer consisting of 10 mM Tris pH 8.0 and 150 mM NaCl. SYPRO Orange was used at a final concentration of 5x, prepared from a 5000x stock in DMSO according to the manufacturer’s instructions.

Each 20 μl reaction mixture was prepared in a white 96-well PCR plate by combining protein, buffer, dye and ADPr (final concentrations of 0 mM or 1 mM). Samples were mixed gently, sealed with optical film, and briefly centrifuged to eliminate bubbles. All reactions were performed in technical triplicate.

Thermal denaturation was monitored using a CFX96 Real-Time PCR Detection System (Bio-Rad). The temperature was increased from 4°C to 70°C at a rate of 1°C/min, and fluorescence was measured every minute using the FRET channel. The melting temperature (T_m_) was determined by uploading the data to DSF World using fitting model 2^66^. Data was plotted using GraphPad Prism.

### Growth curves

Sub-confluent mammalian (BHK-21, Vero, and A549) and mosquito (U4.4) cells in six-well plates were infected with equal amounts of wild-type (WT) or mutant CHIKVs recovered from Vero cells. Infections were performed using a multiplicity of infection (MOI) of 0.05 in 500 μL of phosphate-buffered saline (PBS). One hour post-infection, cells were washed three times with PBS, and 2 mL of growth media were added. At predetermined time points, cell culture supernatants were collected and stored at −80°C. Virus quantification was performed by plaque assay on Vero cells using serial dilutions of the supernatants. To assess mutations/reversions in nsP3 macrodomain viral RNA, cell supernatants from the 48–72 hour time points of growth curves were extracted using TRIzol, amplified by RT-PCR, and analyzed by Sanger sequencing as previously described.

### Mosquito rearing

Laboratory colonies of *Ae. aegypti* mosquitoes (17th generation; collected originally in Kamphaeng Phet Province, Thailand) and *Ae. albopictus* (19th generation; collected originally in Phu Hoa, Binh Duong Province, Vietnam) were used. The insectary conditions for mosquito maintenance were 28°C, 70% relative humidity, and a 12-h-light and 12-h-dark cycle. Adults were maintained with permanent access to a 10% sucrose solution. Adult females were offered commercial rabbit blood (BCL, Boisset-Saint-Priest, France) twice a week through a membrane feeding system (Hemotek Ltd.).

### Ethics

Human blood samples to prepare mosquito artificial infectious bloodmeals were supplied by healthy adult volunteers at the ICAReB biobanking platform (BB-0033–00062/ICAReB platform/Institut Pasteur, Paris/BBMRI AO203/[BIORESOURCE]) of the Institut Pasteur in the CoSImmGen and Diagmicoll protocols, which had been approved by the French Ethical Committee Ile-de-France I. The Diagmicoll protocol was declared to the French Research Ministry under reference 343 DC 2008–68 COL 1. All adult subjects provided written informed consent.

### Experimental infections of mosquitoes

Infection assays were performed with 7- to 10-day-old females starved 24 h prior to infection in a biosafety level 3 (BSL-3) laboratory. Mosquitoes were offered the infectious blood meal for 30 min through a membrane feeding system (Hemotek Ltd.) set at 37°C with a piece of desalted pig intestine as the membrane. The blood meal was composed of washed human erythrocytes resuspended in phosphate-buffered saline mixed 2:1 with a prediluted viral stock and supplemented with 10 mM ATP (Sigma-Aldrich). The viral stock was prediluted in Leibovitz L-15 medium with 0.1% sodium bicarbonate (Gibco) to reach an infectious titer ranging from 1 × 106 to 1 × 10^7^ focus-forming units. Following the blood meal, fully engorged females were selected and incubated at 28°C with 70% relative humidity and under a 12-h-light and 12-h-dark cycle with permanent access to 10% sucrose. At different times postinfection, mosquitoes were cold anesthetized for dissection intro bodies and heads. Body parts were homogenized in microtubes containing steel beads (5-mm diameter) and 300 μl of DMEM supplemented with 2% FBS using a TissueLyser II instrument (Qiagen) at 30 shakes/s for 2 min. Homogenates were clarified by centrifugation and stored at minus 80°C until further processing. Viral titers in individual samples were determined by plaque assays. For the assessment of mutations in nsP3 macrodomain, RNA TRIzol extracted from homogenates was used for RT-PCR and Sanger sequencing as previously described.

## Supplementary Material

Supplement 1

Supplement 2

Supplement 3

Supplemental information

Document S1. Figures S1–S7, Table S1 and supplemental references.

## Resource availability

### Materials availability

Plasmids generated in this study are available from the lead contact upon reasonable request.

### Data and code availability

All data reported in this paper is contained within the main text, figures, and supplementary materials. Crystallographic coordinates and structure factors have been deposited in the PDB with the following accessing codes: 9YHC, 9YHD, 9YHE, 9YHF, 9YHG, 9YHH, 9YHI, 9YHJ, 9YHK, 9YHL and 9YHM.
