# Supplementary material for: Macrodomain ADP-ribose binding but not ADP-ribosylhydrolase activity is critical for chikungunya virus infection of Aedes mosquitoes
Source: bioRxiv. 2025 Oct 9:2025.10.09.681498. Preprint. [Version 1] doi: 10.1101/2025.10.09.681498 (PMC12632316; doi:10.1101/2025.10.09.681498)
Supplement: Supplement 1 [file media-1.pdf]

Supplementary Materials for

Macrodomain ADP-ribose binding but not ADP-ribosylhydrolase activity is critical for chikungunya virus infection of *Aedes* mosquitoes

Eugenia S. Bardossy[1§], Lena Bergmann[2§], Annabelle Henrion-Lacritick[1], Jared Nigg[1†], Galen J. Correy[2], Alan Ashworth[3], James S. Fraser[2*], Maria-Carla Saleh[1*]

1 Viruses and RNAi Unit, Institut Pasteur, Université Paris Cité, F-75015 Paris, France.

2 Department of Bioengineering and Therapeutic Sciences, University of California San Francisco, San Francisco, CA 94158.

3 Helen Diller Family Comprehensive Cancer Center, University of California San Francisco, San Francisco, California, USA.

§These authors contributed equally to this work

†Current address: Chan Zuckerberg Biohub, San Francisco, CA 94158.

*Corresponding authors: jfraser@fraserlab.com; carla.saleh@pasteur.fr

Supplementary Figure 1

a

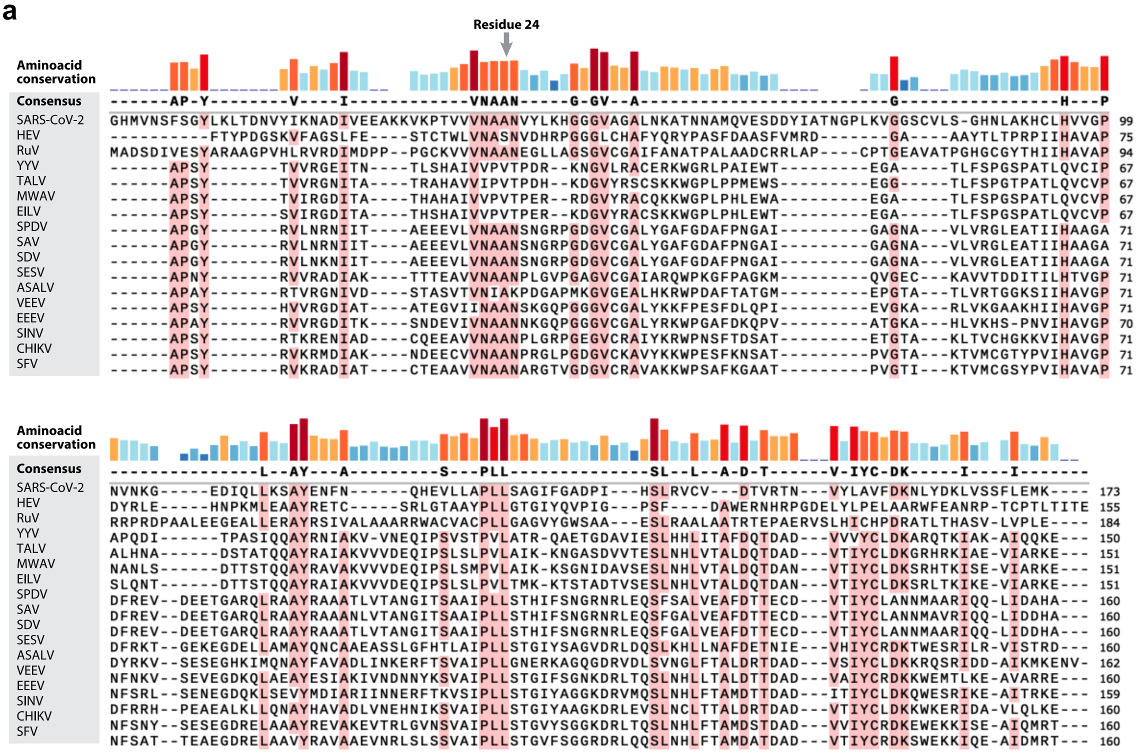

b

Virus acronym	Virus full name	Protein sequence ID	Database
SARS-CoV-2	Severe acute respiratory syndrome coronavirus 2	6YWK_A	PDB
HEV	Hepatitis E virus	NP_056779.1	GenBank
RuV	Rubella virus	8P0C_A	PDB
YYV	Yada yada virus	QGR15362.1	GenBank
TALV	Tai Forest alphavirus	YP_009333615	GenBank
MWAV	Mwinilunga alphavirus	BBC45634.1	GenBank
EILV	Eilat virus	QBG67155.1	GenBank
SPDV	Salmon pancreas disease virus	NP_740637.1	GenBank
SAV	Salmonid alphavirus subtype 3	AGT42235.1	GenBank
SDV	Sleeping disease virus	NP_740655.1	GenBank
SESV	Southern elephant seal virus	YP_005351234.1	GenBank
ASALV	Agua Salud alphavirus	QEV83787.1	GenBank
VEEV	Venezuelan equine encephalitis virus	3GQE_A	PDB
EEEV	Eastern equine encephalitis virus	NP_740651.1	GenBank
SINV	Sindbis virus	4GUA_A	PDB
CHIKV	Chikungunya virus	6VUQ_A	PDB
SFV	Semiliki Forest virus	NP_740667.1	GenBank

Supplementary Figure 1. Conservation of residue 24 among viral macrodomains. a, Multiple sequence alignment of 17 macrodomain protein sequences, including 14 alphaviruses with diverse host ranges (dual-host, mosquito-specific, and aquatic) and 3 other human pathogens encoding viral macrodomains. Amino acid conservation and the consensus sequence are depicted above. Amino acid residues conserved in 80% or more of the analyzed sequences are highlighted in pink, with the corresponding residue indicated in the consensus sequence. Residue 24 is marked with an arrow. **b**, Table listing the full names of viruses included in the alignment, their protein sequence identifiers, and sequence sources.

Supplementary Figure 2

a

Viral stocks (Caribbean)

Virus	Cell line	Timepoint	Position 24 amino acid	Position 24 nucleotides	Position 31 amino acid	Position 31 nucleotides
WT	Vero	1 dpt (P0)	N	aac	D	gac
		1 dpi (P1)	N	aac	D	gac
		2 dpi (P1)	N	aac	D	gac
N24A	Vero	1 dpt (P0)	A	gcc	N	aac
		1 dpi (P1)	A	gcc	N	aac
		2 dpi (P1)	A	gcc	N	aac
N24D	Vero	1 dpt (P0)	D	gac	H/N	cac/aac
		1 dpi (P1)	D	gac	H/N	cac/aac
		2 dpi (P1)	D	gac	H/N	cac/aac

b

Viral stocks after transfection

Virus	Cell line	Timepoint	Position 24 amino acid	Position 24 nucleotides	Position 31 amino acid	Position 31 nucleotides
N24A	A549	0 dpt (P0)	-	-	-	-
		1 dpt (P0)	-	-	-	-
		2 dpt (P0)	A	gcc	D	gac
		3 dpt (P0)	A	gcc	D	gac
		1 dpi (P1)	A/N	gcc/aac	D	gac
	BHK-21	0 dpt (P0)	A	gcc	D	gac
		1 dpt (P0)	A	gcc	D	gac
		2 dpt (P0)	A	gcc	D	gac
		3 dpt (P0)	A	gcc	D	gac
		1 dpi (P1)	A	gcc	D/N	gac/aac
	Vero	0 dpt (P0)	A	gcc	D	gac
		1 dpt (P0)	A	gcc	D	gac
		2 dpt (P0)	A	gcc	D/N	gac/aac
		3 dpt (P0)	A	gcc	D/N	gac/aac
		1 dpi (P1)	A	gcc	N	aac
	C6/36	0 dpt (P0)	A	gcc	D	gac
		1 dpt (P0)	A	gcc	D	gac
		2 dpt (P0)	A	gcc	D	gac
		3 dpt (P0)	-	-	-	-
		1 dpi (P1)	A	gcc	D/N	gac/aac
N24D	A549	0 dpt (P0)	D	gac	D	gac
		1 dpt (P0)	D	gac	D	gac
		2 dpt (P0)	D	gac	D	gac
		3 dpt (P0)	D	gac	D	gac
		1 dpi (P1)	D/N	gac/aac	D	gac
	BHK-21	0 dpt (P0)	D	gac	D	gac
		1 dpt (P0)	D	gac	D	gac
		2 dpt (P0)	D/N	gac/aac	D/N	gac/aac
		3 dpt (P0)	D/N	gac/aac	D/N	gac/aac
		1 dpi (P1)	D/N	gac/aac	D/N	gac/aac
	Vero	0 dpt (P0)	D	gac	D	gac
		1 dpt (P0)	-	-	-	-
		2 dpt (P0)	D/N	gac/aac	D/N	gac/aac
		3 dpt (P0)	D/N	gac/aac	D/N	gac/aac
		1 dpi (P1)	D/N	gac/aac	D/N	gac/aac
	C6/36	0 dpt (P0)	D	gac	D	gac
		1 dpt (P0)	D	gac	D	gac
		2 dpt (P0)	D	gac	D	gac
		3 dpt (P0)	D	gac	D	gac
		1 dpi (P1)	D	gac	D	gac

c

Viral stocks (Indian Ocean)

Virus	Cell line	Timepoint	Position 24 amino acid	Position 24 nucleotides	Position 31 amino acid	Position 31 nucleotides
WT	BHK-21	1 dpt (P0)	N	aac	D	gac
		3 dpt (P0)	N	aac	D	gac
		3 dpi (P1)	N	aac	D	gac
	Vero	3 dpi (P2)	N	aac	D	gac
N24A	BHK-21	1 dpt (P0)	A	gcc	D	gac
		3 dpt (P0)	A	gcc	D	gac
		3 dpi (P1)	A	gcc	D/N	gac/aac
	Vero	3 dpi (P2)	A	gcc	D/N	gac/aac
N24D	BHK-21	1 dpt (P0)	D	gac	D	gac
		3 dpt (P0)	D	gac	N	aac
		3 dpi (P1)	D	gac	N	aac
	Vero	3 dpi (P2)	D	gac	N	aac

Supplementary Figure 2. Sequence analyses of mutations in the CHIKV macrodomain. a, Summary of amino acid and nucleotide variations at residues 24 and 31 in the macrodomain for WT and mutant viruses in the context of the Caribbean CHIKV strain. Viral RNAs of WT, N24A, and N24D variants were transfected into Vero cells, with cell culture supernatants collected and passaged once. Viral stocks were harvested one day after transfection (P0) and at different time points after infection (P1). Total RNA was extracted from viral stocks, followed by reverse transcription-PCR (RT-PCR) of the nsP3 viral gene and Sanger sequencing. **b**, Analysis of macrodomain mutations after transfection of N24A and N24D viral RNAs across mammalian (A549, BHK-21, and Vero) and mosquito (C6/36) cell lines. **c**, Summary of macrodomain mutations in viral stocks of WT and N24 mutant viruses in the context of the Indian Ocean CHIKV strain. Double peaks indicating the coexistence of multiple nucleotides at the same position are denoted by '/'. Amino acid abbreviations: N, asparagine; A, alanine; D, aspartic acid; H, histidine.

Supplementary Figure 3

a

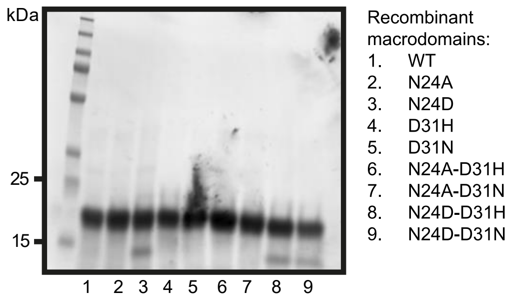

b

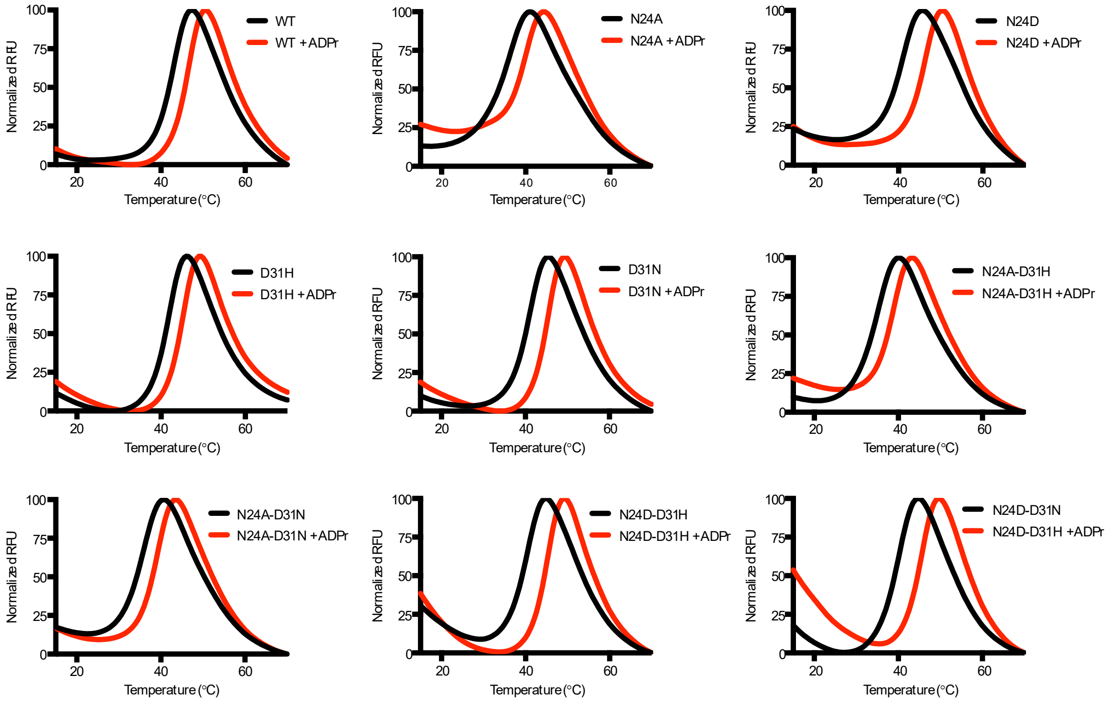

Supplementary Figure 3. Expression of recombinant macrodomains and ADP-ribose binding assays. a, SDS-PAGE analysis of CHIKV nsP3 macrodomain mutants recombinantly expressed and purified from *E. coli*. **b**, Plot showing change in relative fluorescence upon incubation of CHIKV nsP3 macrodomain mutants with SYPRO orange.

Supplementary Figure 4

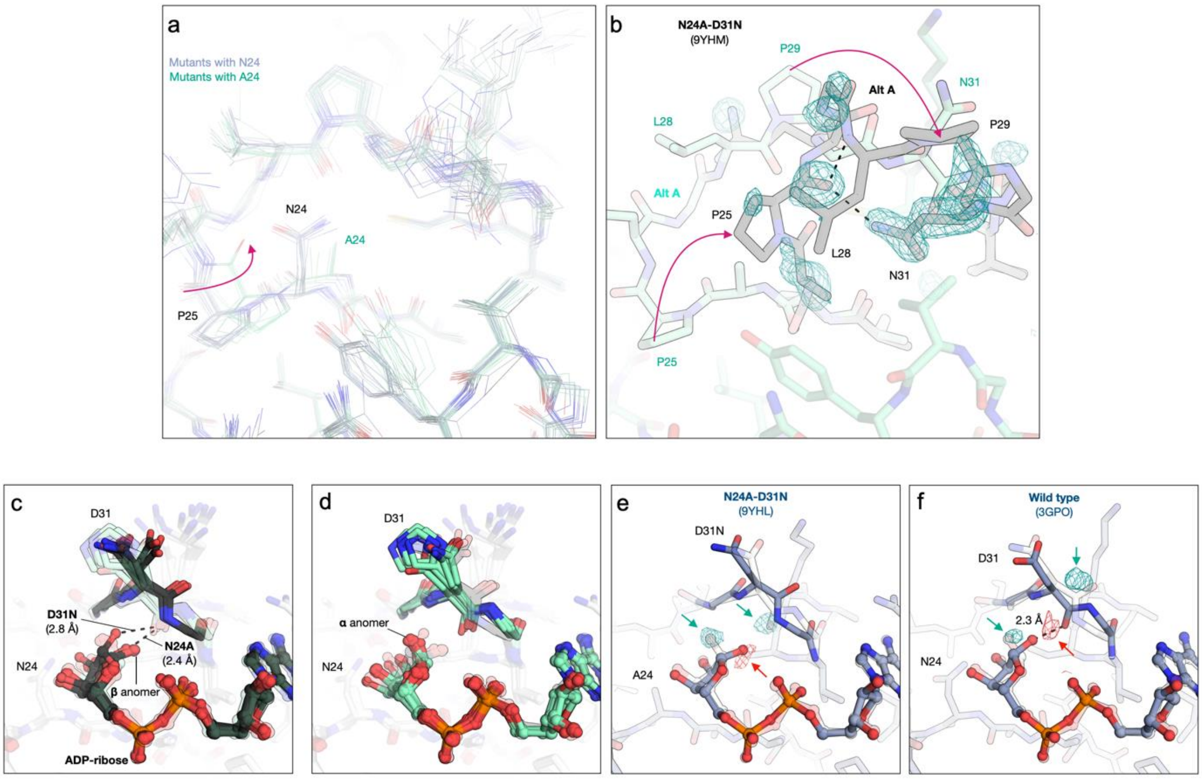

Supplementary Figure 4. Conformational and composition heterogeneity in the CHIKV nsP3 macrodomain. a, Alignment of all apo structures colored by the residue at position 24 (Asparagine = blue, alanine = green). The peptide flip in P25 is observed in some, but not all, of the structures with A24. **b,** Residues 23-33 undergo a large conformational change in chain D of the N24A-D31N structure (PDB 9YHM). The difference electron density map (F_O-F_C, 3 σ) prior to modeling the alternative conformation is shown. **c**, Alignment of all CHIKV nsP3 macrodomain mutant structures in complex with ADP-ribose. The β and α-anomers of ADP-ribose are shown with black and green sticks respectively. The conformation of residue 31 compatible with the β-anomer is shown with black sticks, while the clashing conformation is shown with green sticks. This pattern was not observed for the structures of N24A (PDB 9YHH, chain D) and D31N (PDB 9YHD, chain D) in complex with ADP-ribose, where the β-anomer binds with the N31 carbonyl in the buried conformation. **d**, Same as **c**, but with inverted transparency. **e**, The difference electron density map (F_O-F_C, ± 3 σ) is shown prior to modeling the α-anomer of ADP-ribose or the buried conformation of N24A-D31N (PDB 9YHL, chain D). **f**, The difference electron density map (F_O-F_C, ± 3 σ) calculated from the deposited structure factor amplitudes of a previously reported structure of CHIKV nsP3 macrodomain in complex with ADP-ribose (PDB 3GPO)[17] reveals peaks that are consistent with both adp-ribose anomers and both D31 conformations.

Supplementary Figure 5

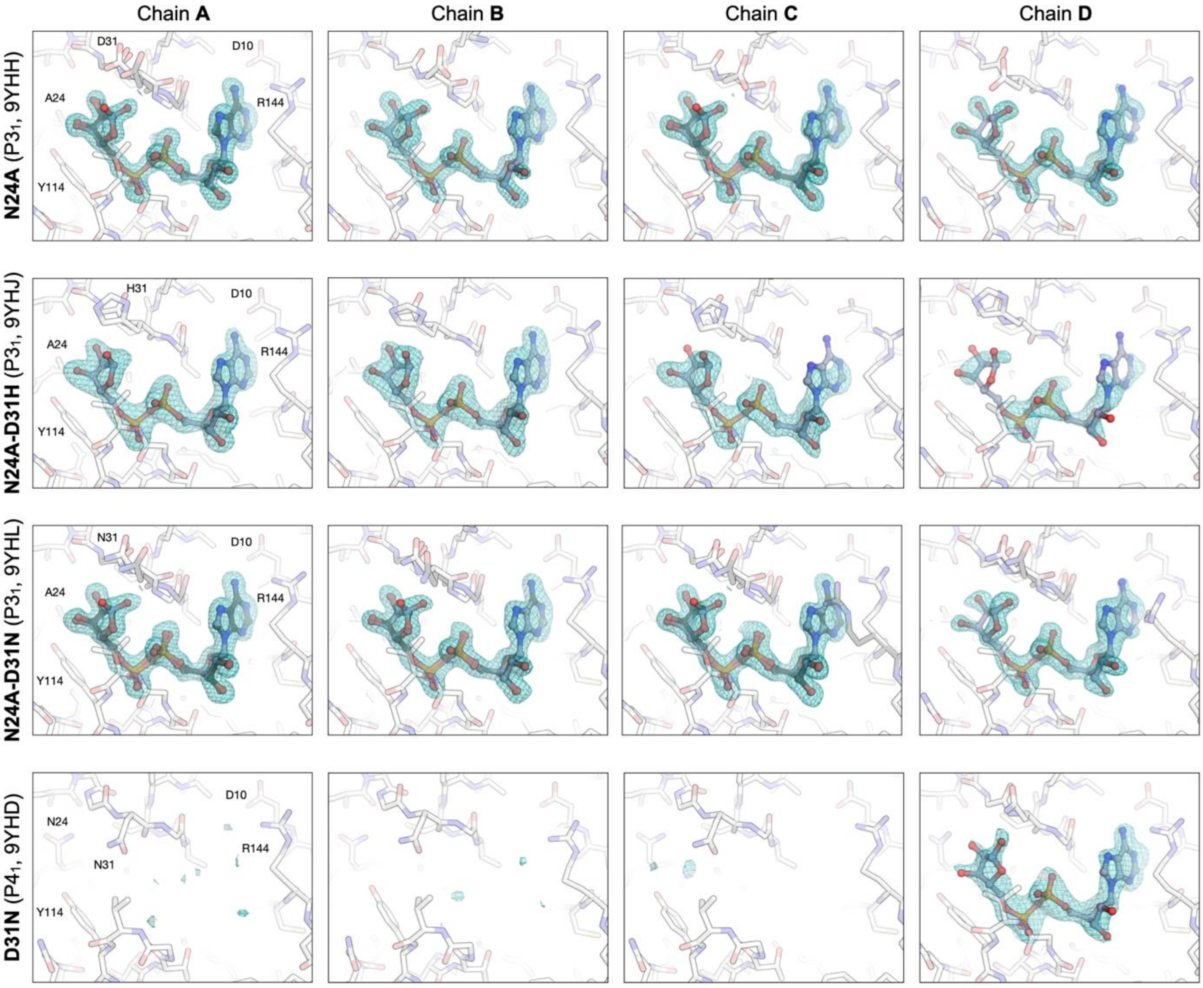

Supplementary Figure 5. Structures of CHIKV nsP3 macrodomain mutants in complex with ADP-ribose. Difference electron density maps (F_O-F_C, 3 σ) calculated prior to ADP-ribose placement for the four structures of CHIKV nsP3 macrodomain determined in complex with ADP-ribose. In the D31N structure, ADP-ribose was only observed in chain D.

Supplementary Figure 6

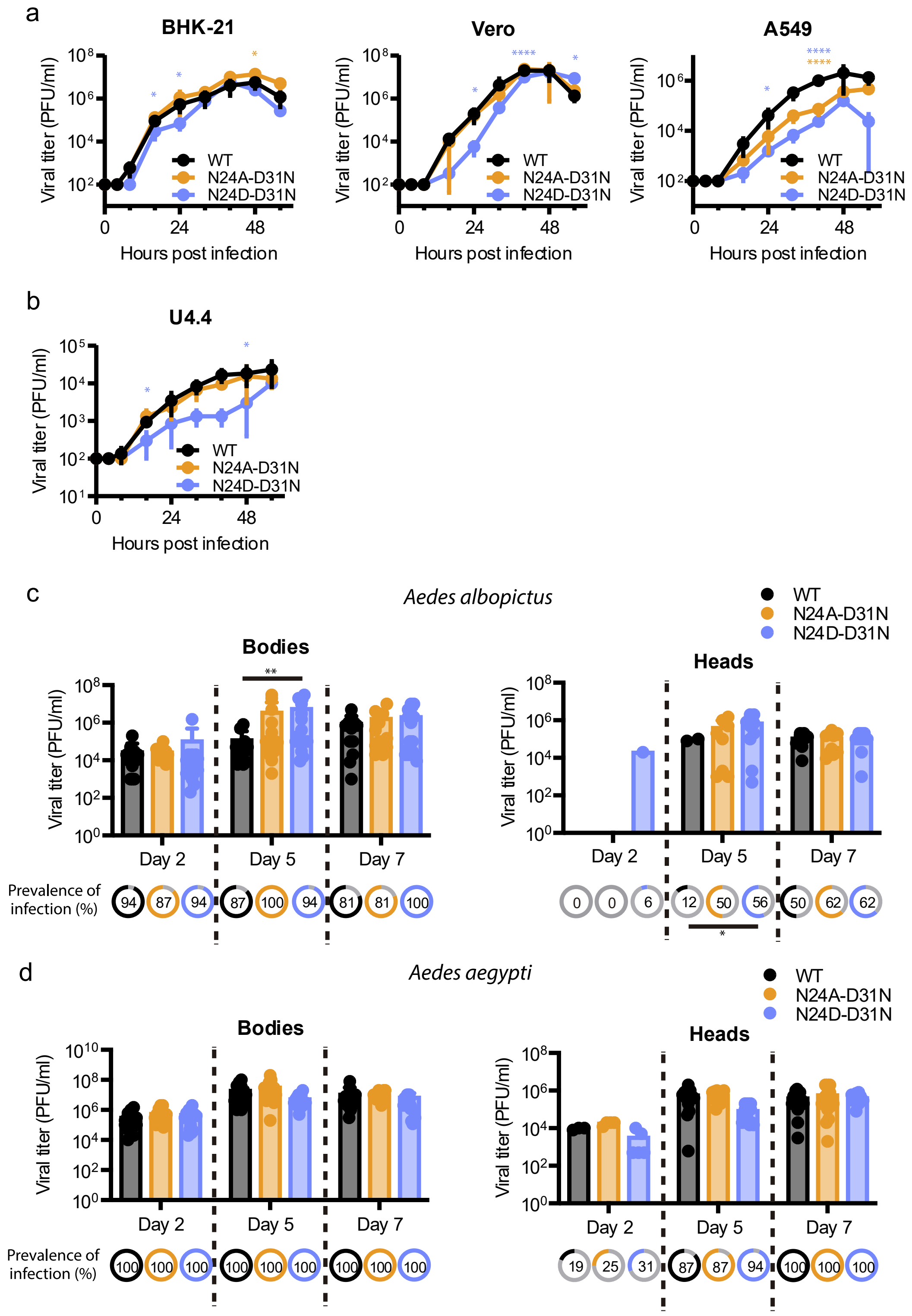

Supplementary Figure 6. Effect of mutations in the macrodomain on Indian Ocean CHIKV replication in mammalian and mosquito cells and in *Aedes* mosquitos. a, Growth kinetics of Indian Ocean WT and mutant viruses (N24A-D31N and N24D-D31H/N) in BHK-21, Vero, and A549 mammalian cells, and in **b**, mosquito U4.4 cells. Error bars represent standard deviations from the mean. n = 3 for 0, 4, 8, 16, 32, 40 and 56 hours post infection (hpi), and n = 6 for 24 and 48 hpi. Data were analyzed using a mixed-effects model with the Geisser-Greenhouse correction followed by a

Tukey's multiple comparison test. Pink and blue asterisks indicate p-values in comparison to WT. **c** and **d**, Laboratory colonies of *Aedes albopictus* and *Aedes aegypti* were exposed to a blood meal containing WT, N24A-D31N (in yellow) or N24A-D31H/N (in blue) viruses. After 2, 5 and 7 days, individual mosquitoes were collected for dissection. Plaque assays were performed on heads and bodies. Viral titers and prevalence of infection in the bodies (left panel) and heads (right panel) of *Ae. albopictus* (**c**) and *Ae. aegypti* mosquitoes (**d**) are shown. 16 mosquitoes were analyzed in all conditions and time points. Error bars represent standard deviations from the mean. Viral titer data were analyzed using a two-way ANOVA followed by a Tukey's multiple comparison test. Prevalences were compared using Fisher's exact test.

Supplementary Figure 7

a

Growth curves (Caribbean)

			Position 24		Position 31	
Virus	Cell line	Timepoint	amino acid	nucleotides	amino acid	nucleotides
WT	BHK-21	48 hpi	N	aac	D	gac
	Vero	48 hpi	N	aac	D	gac
	A549	48 hpi	N	aac	D	gac
	U4.4	48 hpi	N	aac	D	gac
N24A	BHK-21	48 hpi	A	gcc	N	aac
	Vero	48 hpi	A	gcc	N	aac
	A549	48 hpi	A/T	gcc/acc	N	aac
	U4.4	48 hpi	A	gcc	N	aac
N24D	BHK-21	48 hpi	D	gac	H/N	cac/aac
	Vero	48 hpi	D	gac	H/N	cac/aac
	A549	48 hpi	D	gac	H/N	cac/aac
	U4.4	48 hpi	D	gac	H/N	cac/aac

b

Growth curves (Indian Ocean)

			Position 24		Position 31	
Virus	Cell line	Timepoint	amino acid	nucleotides	amino acid	nucleotides
WT	BHK-21	72 hpi	N	aac	D	gac
	Vero	72 hpi	N	aac	D	gac
	A549	72 hpi	N	aac	D	gac
	U4.4	72 hpi	N	aac	D	gac
N24A	BHK-21	72 hpi	A	gcc	N	aac
	Vero	72 hpi	A	gcc	N	aac
	A549	72 hpi	A	gcc	N	aac
	U4.4	72 hpi	A	gcc	D/N	gac/aac
N24D	BHK-21	72 hpi	D	gac	N	aac
	Vero	72 hpi	D	gac	N	aac
	A549	72 hpi	D	gac	N	aac
	U4.4	72 hpi	D	gac	N	aac

Supplementary Figure 7. Sequence analyses of mutations in the CHIKV macrodomain from viral stocks recovered during growth curves. a, Summary of amino acid and nucleotide variations at residues 24 and 31 in the macrodomain of Caribbean WT and mutant viruses during growth curves shown in Figure 4. Viral stocks were collected 48 hours post-infection, and total RNA was extracted, followed by reverse transcription-PCR (RT-PCR) of the nsP3 viral gene and Sanger sequencing. **b**, Analysis of macrodomain mutations in Indian Ocean WT and mutant viruses during growth curves shown in Supplementary Figure 4. Double peaks indicating the coexistence of multiple nucleotides at the same position are denoted by '/'.

Supplementary Table 1. Thermostability and ADP-ribose binding of WT and mutant macrodomains measured by differential scanning fluorimetry (DSF). Data are reported mean ± SD for three technical replicates.

	**Tm**	**+ 1mM ADPr**	**ΔT_m**
WT	43.2 ± 0.1	46.5 ± 0.1	3.2 ± 0.1
N24A	35.9 ± 0.4	40.0 ± 0.1	4.1 ± 0.4
N24D	41.3 ± 0.2	46.1 ± 0.2	4.7 ± 0.2
D31H	41.9 ± 0.2	45.1 ± 0.3	3.2 ± 0.4
D31N	41.3 ± 0.3	45.4 ± 0.1	4.1 ± 0.3
N24A-D31H	35.3 ± 0.4	39.1 ± 0.6	3.8 ± 0.7
N24A-D31N	36.2 ± 0.4	39.4 ± 0.1	3.2 ± 0.4
N24D-D31H	40.7 ± 0.3	45.6 ± 0.5	4.9 ± 0.6
N24D-D31N	40.4 ± 0.1	45.6 ± 0.2	5.1 ± 0.2

Supplementary Table 2. X-ray data collection and refinement statistics. (Supplementary_Table_2.xlsx)

Supplementary Table 3. Sequence analysis of CHIKV macrodomain mutations recovered from viral RNA in individual *Aedes* mosquitoes. (Supplementary_Table_3.xlsx)
